# The Holstein Friesian Lethal Haplotype 5 (HH5) Results from a Complete Deletion of *TBF1M* and Cholesterol Deficiency (CDH) from an ERV-(LTR) Insertion into the Coding Region of *APOB*

**DOI:** 10.1371/journal.pone.0154602

**Published:** 2016-04-29

**Authors:** Ekkehard Schütz, Christin Wehrhahn, Marius Wanjek, Ralf Bortfeld, Wilhelm E. Wemheuer, Julia Beck, Bertram Brenig

**Affiliations:** 1 Institute of Veterinary Medicine, Georg-August-University Göttingen, Göttingen, Germany; 2 Institute for Livestock Reproduction GmbH, Schönow, Germany; 3 Chronix Biomedical GmbH, Göttingen, Germany; CSIRO, AUSTRALIA

## Abstract

**Background:**

With the availability of massive SNP data for several economically important cattle breeds, haplotype tests have been performed to identify unknown recessive disorders. A number of so-called lethal haplotypes, have been uncovered in Holstein Friesian cattle and, for at least seven of these, the causative mutations have been identified in candidate genes. However, several lethal haplotypes still remain elusive. Here we report the molecular genetic causes of lethal haplotype 5 (HH5) and cholesterol deficiency (CDH). A targeted enrichment for the known genomic regions, followed by massive parallel sequencing was used to interrogate for causative mutations in a case/control approach.

**Methods:**

Targeted enrichment for the known genomic regions, followed by massive parallel sequencing was used in a case/control approach. PCRs for the causing mutations were developed and compared to routine imputing in 2,100 (HH5) and 3,100 (CDH) cattle.

**Results:**

HH5 is caused by a deletion of 138kbp, spanning position 93,233kb to 93,371kb on chromosome 9 (BTA9), harboring only dimethyl-adenosine transferase 1 (*TFB1M*). The deletion breakpoints are flanked by bovine long interspersed nuclear elements Bov-B (upstream) and L1ME3 (downstream), suggesting a homologous recombination/deletion event. TFB1M di-methylates adenine residues in the hairpin loop at the 3’-end of mitochondrial 12S rRNA, being essential for synthesis and function of the small ribosomal subunit of mitochondria. Homozygous *TFB1M*^-/-^ mice reportedly exhibit embryonal lethality with developmental defects. A 2.8% allelic frequency was determined for the German HF population. CDH results from a 1.3kbp insertion of an endogenous retrovirus (ERV2-1-LTR_BT) into exon 5 of the *APOB* gene at BTA11:77,959kb. The insertion is flanked by 6bp target site duplications as described for insertions mediated by retroviral integrases. A premature stop codon in the open reading frame of *APOB* is generated, resulting in a truncation of the protein to a length of only <140 amino acids. Such early truncations have been shown to cause an inability of chylomicron excretion from intestinal cells, resulting in malabsorption of cholesterol. The allelic frequency of this mutation in the German HF population was 6.7%, which is substantially higher than reported so far. Compared to PCR assays inferring the genetic variants directly, the routine imputing used so far showed a diagnostic sensitivity of as low as 91% (HH5) and 88% (CDH), with a high specificity for both (≥99.7%).

**Conclusion:**

With the availability of direct genetic tests it will now be possible to more effectively reduce the carrier frequency and ultimately eliminate the disorders from the HF populations. Beside this, the fact that repetitive genomic elements (RE) are involved in both diseases, underline the evolutionary importance of RE, which can be detrimental as here, but also advantageous over generations.

## Introduction

Reproductive performance of high-yielding dairy cattle especially Holstein Friesian (HF) has gradually declined since around the 1980s [1]. However, due to an increased attention for these unfavorable developments, many countries started recording reproductive parameters and included fertility traits in their national breeding programs [2–4]. Unfortunately, reproductive performance of a breed is a complex trait depending on a variety of factors including male and female fertility, animal health, herd management, environment, feeding as well as calving traits. Hence, heritability for reproductive performance is usually extremely low in cows (0.02 to 0.04) and only slightly higher in sires (0.05 to 0.22) [5]. Despite these limitations, the recent introduction of genomic selection tools into classical breeding programs has already helped to improve some of the adverse trends [5–8]. According to the quantitative survey of the Council on Dairy Cattle Breeding for example, daughter pregnancy rate, cow conception rate and sire conception rate steadily increased over the last ten years in the US (www.cdcb.us/eval.htm). This encouraging development should however not belie the fact that many functional traits are negatively correlated with milk yield [1].

In recent years it was extremely helpful that molecular biological techniques became available that allowed high through-put genotyping and deep sequencing of livestock. These techniques facilitated the decipherment of numerous genetic and physiological mechanisms responsible for the decline in reproductive performance [9]. Whole-genome haplotyping has been applied to identify recessive disorders affecting fertility and stillbirth [10, 11]. In 2011, three lethal haplotypes affecting fertility in HF were identified located on bovine chromosomes 1 (HH1), 5 (HH2) and 8 (HH3) and the heterozygous founders were traced back [11]. Two years later, 14 additional lethal haplotypes (HH4-HH17) were reported in HF and meanwhile three were resolved on the molecular level, *i*.*e*. HH1 (*APAF1*), HH4 (*GART*) and HH5/HH6 (*SLC35A3*) [12]. A mutation in *SMC2* was shown to be causative for lethal haplotype HH3 [13]. Several lethal haplotypes with non-negligible frequencies including HH2 (BTA1) and HH5 (BTA9) still await further clarification.

Only recently, it was reported that homozygous calves of an additional lethal haplotype in HF, *i*.*e*. CD (cholesterol deficiency, CDH), showed markedly reduced cholesterol serum levels [14], and was traced back to the sire Maughlin Storm, born in 1991. Further clinical signs normally manifesting in homozygous calves between 1–5 months of age include decreased appetite, weight loss, diarrhea with unclear etiology, and eventually death. The lethality rate of homozygous CDH carriers was 80% and the reported carrier frequency in the HF population was 8.7%. A genome wide association study re-adjusted the chromosomal region on bovine chromosome 11 (BTA11) from the initially given position 76.4–79.9 Mb [15] to positions 74.5–77.0 Mb [14]. Unfortunately, the latter region of the bovine genome is not yet well annotated and therefore a direct search for potential candidate genes was impeded. However, in humans, diseases with similar clinical signs have been described, *e*.*g*. Anderson disease (chylomicron retention disease) and familial hypobetalipoproteinemia (FHBL1) [16–20]. Anderson disease is caused by mutations in *SAR1B* on HSA5q31.1. Although *SAR1B* was shown to be associated with milk production traits in cattle, it was excluded as candidate gene due to its location on BTA7 [21]. On the other hand genetically truncated apolipoprotein-B (APOB) proteins have been associated with this disease more than 30 years ago [22, 23] and were linked to haplotypes around the *APOB* gene in family analyses [24]. Therefore, *APOB* on HSA2p24.1 involved in the development of FHBL1 was a likely candidate gene because in cattle it is located on BTA11 between positions 77.9–78.0 Mb within the region, initially found to be associated with CDH. None of the other approximately 29 genes located within this chromosomal region could be functionally linked with the clinical signs in a meaningful way. The recently published region of BTA11:74.5–77.0 Mb [14], would exclude *APOB*. Nevertheless, given the clinical signs of affected cattle and the reported biochemical findings, the reliability of these data seemed questionable and *APOB* remained the most likely candidate gene for CDH.

The second most important haplotype is HH5 [15], which has an assessed heterozygous carrier frequency of approximately 4–5% in the European and North American HF population and results in a premature pregnancy termination before day 60 of gestation. The genomic region that defines the respective haplotype was reported to be located around 91.8–93.8Mb on BTA9 (http://www.holsteinusa.com/news/press_release2013.jsp#pr2013_20) and was traced back to Thornlea Texal Supreme born in 1957. The underlying causative genetic mutation was unknown and in contrast to CDH no obvious candidate gene was present in the associated chromosomal region.

Using a strategy of selective enrichment of the said regions for the two haplotypes was used, followed by high throughput sequencing in order to unveil genomic variants that segregate with the haplotypes.

## Materials and Methods

### Ethical statement

Blood samples were collected during routine diagnostic parentage control and genomic selection performed with written owner consent. Blood samples were drawn exclusively by local veterinarians. Blood sampling by veterinarians with state examination is in accordance with the German Animal Welfare Act (§6 Abs. 1 Satz 2 TierSchG). Therefore no formal ethical approval was required, since no other samples were collected for this study.

### Animals

For CDH, a total of 46 descendants of sire Maughlin Storm (MS) were selected and subjected to SNP genotyping with the Illumina BovineSNP50 BeadChip. Haplotypes were imputed from our in-house database of about 30,000 cattle results and the haplotype for cholesterol deficiency was imputed using Beagle 4.1 [25]. In addition, 11 homozygous animals were selected from the same data set and their health status was verified. For targeted re-sequencing 29 CD positive, 17 CD negative MS descendants, 9 CD homozygous affected animals, 2 CD homozygous healthy, and 13 random control animals imputed to non-carriers of CDH, were selected.

For HH5, carriers of HH5 were selected from approximately 54,000 HF SNP genotyping data using the Illumina BovineSNP50 BeadChip and deposited at vit (Verden, Germany). All carriers were checked and filtered for available direct offspring with and without carrier status. The resulting 90 sires were tested for relatedness using the 200 ISAG SNPs established for parentage control on an each vs. each basis. From the resulting matrix those with the highest number of mismatches were selected. Together with each chosen sire, offspring with and without imputed carrier status were selected together with the available respective dams. The selection resulted in a total of 33 HH5 carriers. Control animals (n = 60) were selected randomly from the database and were imputed as non-carriers HH5. In total, 93 samples were used for targeted next generation sequencing.

### Target Enrichment

A direct genomic hybridization enrichment using selected bovine BAC clones was conducted as described elsewhere [26]. A total of 42 BAC clones were selected, 16 covering the HH5 (S1 Table) and 26 the CDH region plus the *APOB* locus (S2 Table). BAC DNA was isolated using PhasePrep BAC DNA Kit (Sigma-Aldrich) and biotinylated by nick translation (Nick Translation Kit, Roche). The resulting fragment lengths were ~300bp and ~500bp, respectively.

Extracted bovine genomic DNA was sheared by hydrofocus to sizes of about 150-300bp (Covaris S2). Sequencing adaptors for next generation sequencing were ligated to the sheared DNA using the NEBNext Ultra DNA Library Prep Kit for Illumina (New England Biolabs, Munich, Germany). To enable de-multiplexing after sequencing a unique molecular identifier was used for each animal.

For hybridization, DNA of the 16 or 26 BAC clones was equally pooled to a total amount of 100ng and repeats were suppressed by incubation with bovine Hybloc/Cot-1 DNA (Applied Genetics Laboratories) for six hours at 65°C. Each hybridization reaction was performed with 1μg of ten equally pooled genomic DNA samples. The incubation was carried out for 48h at 65°C, followed by capture of the hybridized fragments with streptavidin beads (Dynabeads M-280 Streptavidin, Invitrogen) and purification using Sephadex-G50 mini Quick Spin Columns (Roche). After the first round of selection, the DNA was amplified, purified (QIAquick PCR Purification Kit, Qiagen), and subjected to a second hybridization reaction as described above. The resulting selected DNA was quantified using a 2100 Bioanalyzer (Agilent Technologies).

### Sequencing and Data Analysis

Sequencing was performed using 2x150bp sequencing on a NextSeq500 platform, the sequences were aligned to the bovine genome build UMD3.1 using BWA and subjected to variant calling using the GATK pipeline [27] performed on a 48cpu Linux computer cluster. The HH5 target region was covered with an average 150-fold base coverage per animal (STD: 78). The CDH target region showed a 50-fold base coverage (STD: 15). To assess copy-number differences the number of matching reads in 1000bp along the targeted regions were counted and normalized by the total number of reads within the target region per animal. The mean normalized read counts were calculated for carriers and controls and for each bin the ratio of mean_carriers_/mean_controls_ was calculated and logarithmized with base 2. Structural variants were inferred by using SVDetect [28] and for short indels, the data were filtered for partially mapping reads (soft clips) and the mapping positions were analyzed for frequency and compared between the groups.

### HH5 Deletion Genotyping with PCR

Real-time duplex PCR with subsequent High Resolution Melting was performed in a total volume of 25 μL with 1 U FastStart*Taq* Polymerase (Roche Diagnostics GmbH), 200 μmol/L of each dNTP (Roche Diagnostics GmbH), 0.2 μmol/L of each reverse primer, 0.4 μmol/L of forward primer (S3 Table), 1 x PCR buffer (incl. 2 mmol/L MgCl_2_, Roche Diagnostics GmbH), 1 x EvaGreen (Jena Bioscience) and 30–50 ng of template DNA. Amplification and analysis was done in a LightCycler 480 (Roche Diagnostics GmbH) using filter set 465–510 with an initial denaturation at 95°C for 10 min, followed by 35 cycles at 95°C for 30 s, 55°C for 30 s, and 72°C for 45 s with a single measurement at the end of each cycle and a final elongation at 72°C for 7 min. Subsequently, High Resolution Melting was done at 95°C for 1 min (4.4°C/s), 40°C for 1 min (2.2°C/s), 70°C for 1 s (1°C/s), an increase to 90°C with the continuous acquisition mode (0.02°C/s), and 40°C for 1 s (2.2°C/s). The samples were genotyped by using the Melt Curve Genotyping Analysis Module of the LightCycler 480 Software (release 1.5.0 SP3, Roche Diagnostics GmbH). When calculating the first negative derivative, the wild-type amplicon and the deletion specific amplicon appeared as clearly distinguishable melting peaks with a T_m_ at 80.5°C and 79.0°C, respectively (S1 Fig).

### CDH Insertion Genotyping with PCR

Real-time duplex PCR with subsequent melting curve analysis was performed as shown for HH5 with 1 μmol/L of insertion specific primers, 0.5 μmol/L of wild-type specific primers (S3 Table), 1 x PCR buffer (incl. MgCl_2_, Roche Diagnostics GmbH), 1 x EvaGreen (Jena Bioscience) and 30–50 ng of template DNA. Amplification and analysis was done in a LightCycler 480 (Roche Diagnostics GmbH) using filter set 465–510 with an initial denaturation at 95°C for 10 min, followed by 33 cycles at 95°C for 15 s, 57°C for 20 s, and 72°C for 30 s with a single measurement at the end of each cycle at 80°C. Subsequently a melting curve was recorded from 80°C to 95°C (5 acquisitions/C°). Melting curves were then evaluated as described for HH5. Each of the two amplicons showed a clear distinct melting peak with a T_m_ at 86.6°C and 88.7°C for the wild-type and insertion specific amplicon, respectively (S2 Fig).

In addition to verify the insertion size, long range PCRs were performed with DNA from affected and unaffected cattle using primers targeting the up- and downstream insertion sides and spanning the insertion sides with ~ 100bp distance each, using the Expand Long Range PCR plus kit (Roche Diagnostics). Agarose gel electrophoresis was performed on samples from affected and unaffected cattle to further characterize the insertion.

### Assessment of in vivo effects

The ratio of mitochondrial (MT) to nuclear DNA was determined with a published droplet digital PCR method [29]. In brief, DNA extracted from whole blood was used, where for MT-DNA 100 pg and for nuclear DNA 40 ng were used in a 20 μL reaction volume. After droplet generation and PCR, droplets were counted in a QX200 droplet reader (BioRad, Munich, Germany). Counting results were converted into copies/reaction, based on Poisson distribution and the ratio was calculated accounting for the dilution of MT-DNA.

Cholesterol plasma concentrations from retained blood samples were measured by an enzymatic/colorimetric routine method (Roche Diagnostics, Mannheim, Germany).

### Statistics

Sensitivity, specificity and accuracy were calculated using accepted common formulas [30]. Confidence intervals for counting statistics based estimation of allelic frequencies were calculated assuming a Poisson distribution (mean equals variance) of observations in random groups at the respective limit of ±1.96 from the Gaussian distribution.

## Results

As a first step, the sequences were interrogated for nucleotide polymorphisms and short insertion/deletions. No mutations that could be attributed to result in effects on annotated proteins were found to segregate with the carrier status of CDH and HH5 haplotypes. The obtained base coverage of 50-fold for CDH and 150-fold for HH5 together with the number of homozygous and heterozygous cattle was sufficiently high to ensure the detection of any potential variant.

### Fine mapping of CDH region

**Fig 1:**
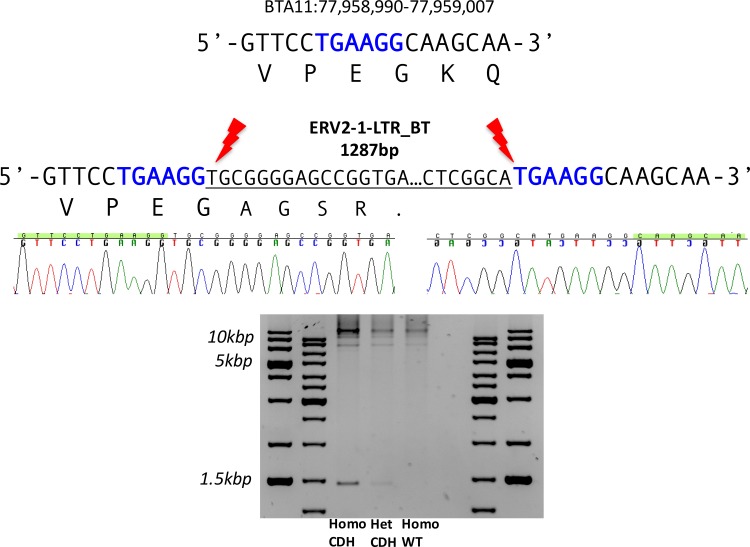
Schematic drawing of the retroviral insertion in *APOB* exon 5 causing CDH. The wind-type sequence and amino acid translation is shown on top (location on BosTaurus_UMD3.1/bosTau6). The insertion breakpoints of the ERV2-1-LTR_BT are shown below. The insertion causes a premature stop codon. The 6bp target site duplication indicative of an integrase mediated insertion is highlighted in blue. The chromatograms are obtained from Sanger-sequencing of a 1,461bp amplicon that was obtained with primers unique to the flanking region of the putative insertion site. As shown in the agarose gel image this band was amplified in CDH homozygous/heterozygous animals but is not present in the wild-type control animal.

No significant large insertion/deletion was detected by copy number analysis in 5kbp bins. In addition no unambiguous variants were detected by using SVdetect. Therefore, to infer for smaller structural variants, we conducted an additional search for positions, were full length (150bp) reads of high sequence quality showed only a partial mapping, with the result of soft-clipping of the reads by BWA. This revealed a highly segregating position in the CDH region (S3 Fig) on BTA11:77,958,995 (UMD3.1), which was seen in 285 reads in the CD group, as compared to one read in controls. The number of reads (±5kb around the position above) was comparable in both groups (230k vs 234k), resulting in an odds-ratio of 290 (CI^95^: 41–2066, p<0.00001). Unmapped pairs of reads mapping ±100bp around that position were assembled, yielding two contigs of ~300bp. These showed the highest sequence identity to an ERV2-1-LTR_BT, flanked by 6bp target site duplications as described for insertions, mediated by retroviral integrases [31, 32]. The 3’ and 5’ ends of the contigs mapped to the upstream and downstream region of the above given insertion point on BTA11. The insertion on BTA11 is located in exon 5 of the *APOB* gene and is resulting in a premature stop codon with the consequence of a truncated protein (Fig 1). The amino acid number of the bovine protein cannot be given precisely, since the full length RNA of bovine *APOB* has not been characterized yet, however, the insertion disrupts the amino acid sequence after the motif KLAVPEG, which corresponds to KLAIPEG at the position aa132-138 in the human protein.

After agarose gel electrophoresis (Fig 1) of products from long range PCRs using primers spanning the insertion side, two bands (1.5 and ~7.5kbp) are visible, of which the 1.5kbp band was only seen in samples from diseased cattle. Both bands were picked and directly investigated for the presence of the insertion by PCR, where only the 1.5kbp band was positive for both breakpoints. The high molecular band was only reactive for the upstream breakpoint PCR, which in addition showed a shifted melting peak. From both bands NGS libraries were constructed and sequenced, revealing a high similarity to ERV-2-1_LTR_BT for the 1.5kb band, whereas the 7.5kbp band did not show a congruent mapping pattern. This suggests a spurious unspecific amplification of an e.g. repetitive element. Using different sets of primers—including those targeting the breakpoints directly—abolished the amplification of that band in most primer combinations. The 1.5kbp band, remaining the only candidate band, was cloned into a pGEM-T vector and grown in bacteria. After extraction of plasmid DNA, the inserts were sequenced on an ABI 3130xl Genetic Analyzer. The assembled sequence revealed an insert size of 1,287bp (including two duplications of 1,299bp) and displayed a 98.5% homology to the consensus sequence of the ERV-2-1-LTR_BT entry in the database of repetitive elements(www.girinst.org) [33].

### In vivo effect verification

Retained plasma samples from 6 homozygous and 10 heterozygous CD animals as well as 10 randomly selected controls used to quantify the cholesterol and concentration in blood plasma. The results in Table 1 show the hypocholesterolemia in affected cattle suggesting a co-dominant effect of the mutation on lipid homeostasis. In the group of heterozygous cases two animals, which were imputed as wild-type, but confirmed to harbor the *APOB* insertion by PCR exerted a cholesterol level of 50 and 46 mg/dL, and therefore are biochemically verified as carriers.

**Table 1:** Lipid status of cattle grouped by CD mutation.

CD mutation	homozygous	heterozygous	wild-type
Cholesterol (mg/dL)	9±3^a^^,^^b^	55±13^b^	125±33

^a)^: p<0.0001 vs heterozygous.

^b)^: p<0.0001 vs wild-type.

### Fine mapping of HH5 region

**Fig 2:**
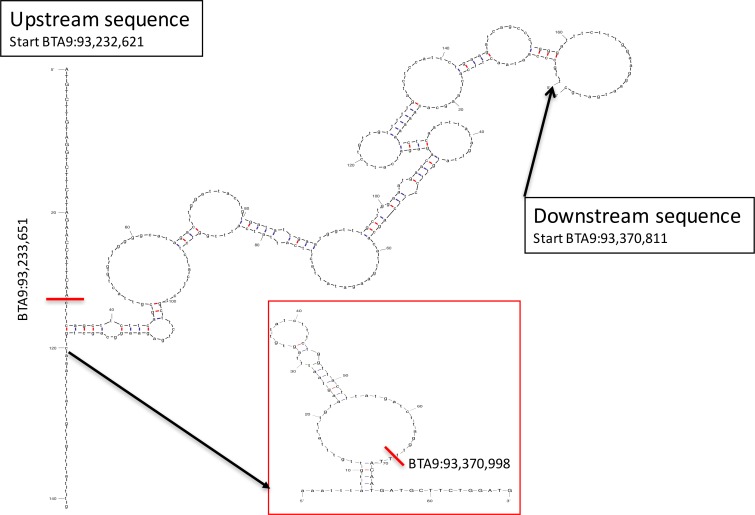
Schematic drawing of the most likely homologous hybridization between the two repetitive elements within the deleted sequence. A stable dsDNA structure is formed with a free Gibbs energy (∆G^37^°) of -13.5 kcal/mol and further downstream a stable secondary structure within the single strand is formed (∆G^37^° = -4.3 kcal/mol), shown as insert. The fusion points are marked with red lines, based retained after fusion of the sequences are shown as capital letters, small letters depict the sequence parts that are deleted. Calculations and drawings are created using UNAfold (http://unafold.rna.albany.edu) [46].

Within the HH5 region, copy number variation analysis revealed an approximately 138kb region spanning 93.233Mb to 93.371Mb suggestive to be deleted in HH5 carriers, which all had a log2 ratio of about -1 in this interval (S4 Fig). The detailed inspection of the sequencing data revealed a breakpoint and fusion between BTA9:93,232,651 and BTA9:93,370,998. At both sides bovine repetitive elements are annotated. At the upstream breakpoint a member of the Bov-B family was located, whereas at the downstream breakpoint a L1ME3 element of the L1 LINE family was present. Fig 2 depicts a potential homologous recombination/deletion mechanism (reviewed in [34]) most likely responsible for this aberration.

Only one reference gene is currently annotated within the deleted region coding for dimethyladenosine transferase 1 isoform 1, or transcription factor B1 mitochondrial (*TFB1M*). TFB1M is critical for the initiation of protein translation in mitochondria explaining the lethal effect in homozygotes. The major function is the dimethylation of adenine residues located in the hairpin loop at the 3’-end of mitochondrial 12S rRNA, which seems to be critical for synthesis and function of the small subunit the ribosome [35, 36]. A loss in 12S rRNA methylation destabilizes ribosomes resulting in loss of mitochondrial protein translation with severe, mostly lethal consequences for cells [37].

### In vivo effect verification

In order to elucidate whether the *TFB1M* deletion has a co-dominant effect in carrier cattle, the ratio of mitochondrial DNA to nuclear DNA was determined by ddPCR in DNA extracted from white blood cells in randomly chosen cattle. There was no significant difference between carriers and wild-type, suggesting a recessive effect, which is in agreement with the phenotype of those animals, which do not show any noticeable abnormalities.

For CDH and HH5 PCRs were designed that cover the insertion and fusion points respectively, to unambiguously interrogate for the two causal variants. For HH5 a total of ~2,300 individuals were analyzed. 189 of these animals harbored the haplotype as inferred from SNP typing and were independent of the previous investigations of searching for the cause HH5. The detection sensitivity by the imputation was only 91.4%, when compared to the diagnostic PCR, whereas false positive imputing was scarce. The allelic frequency in a randomly selected 1980 HF cattle subset was 2.8% (CI^95^: 2.2% - 3.5%) with a calculated carrier frequency of 5.5% (CI^95^: 4.3% - 6.7%).

For CDH, we used 3,100 (preselected and random) cattle, for which the definite PCR assay was compared to results from haplotyping. Overall the imputation system that is used so far was found to yield a rate of 97.8% accuracy in predicting the disease status of CDH, whereas the detection rate of the carriers (n = 419)—on the other hand—was found to be more error prone, with a sensitivity of as low as 88.3%. As a consequence of the high number of false negatives, the allelic frequency of the *APOBins*, when assessed with the PCR method in 2,200 randomly selected cattle (born 2012–2015) increased to 6.7% (CI^95^: 5.6% - 8.0%) in the tested subset, yielding a calculated carrier frequency of 12.7% (CI^95^: 10.5% - 14.6%), as opposed to the initially reported value of approximately 8.7% [14].

Table 2 summarizes the mutation frequencies of the lethal haplotypes in the German HF population. Direct genotyping of the causative genes was performed in approximately 2,000 HF cattle for HH5 and CDH. From about 14,000 datasets from the bovine EuroG10kv4 bead chip, the frequency of HH2 was determined by imputing, the remainders were genotyped with probes interrogating the mutation directly present on the same chip.

**Table 2:** Overview over lethal haplotype frequencies in a subset of the German HF population.

Haplotype	Gene	Chromosomal position	Frequency of carriers (%)
HH1	*APAF1*	5:63,150,400bp [T/C]	1.8
HH2	unknown	1:94.68Mb– 96.47Mb	1.7
HH3	*SMC2*	8:95,410,507bp [T/C]	5.1
HH4	*GART*	1:1,277,227bp [A/C]	4.0
HH5	*TFB1M*	9: 93,233 kb[del]138kb	5.5
CDH	*APOB*	11:77,959kb[Ins]	12.7

### Discrepancy analyses

For both CDH and HH5 those samples for which divergent results between haplotype imputing and the definite PCR was observed were examined to determine the reasons for the differences. For CDH we experienced that 12.4% of affected cattle investigated (n = 307) are yielding a false negative imputing. By inspection of those cases, it was obvious that the majority of false negative imputing results was due to several breaks within the haploblock used. In CDH carriers the breaks observed closest to the *APOBins* position were: upstream between BTA11:77,560,928 and BTA11:77,735,570 as well as downstream between BTA11:78,545,016 and BTA11:78,645,742. As an additional source of errors by haplotyping the goodness of SNP data must be considered. We identified one false negative case as mostly technically, since the genotype calling score of that sample in the CDH region was significantly lower than seen in controls with Z = -4.8 standard deviations (P<0.0001).

One solution for an imputing of CDH (which is important *e*.*g*. for historic data) is to take these recessed haplotypes into account, doing so resulted in lowering the rate of false negatives to ~3–4% for CDH cases found positive in the PCR method with our dataset.

For HH5 the inspection of falsely negative imputed carriers revealed several breaks of the respective haploblock, with the closest to the *TFB1M* deletion between BTA9:92,300,111 and 92,350,052 (upstream) as well as BTA9:93,430,532 and 93,472,326 (downstream). As seen for CDH, one false negative sample in the HH5 region also had a significantly lower average GC-Score compared to controls with Z = -4.9 standard deviations (P<0.0001) and is therefore most likely of technical cause.

## Discussion

The frequent use of certain sires and their descendants in modern cattle breeding is bearing the risk for manifestation of genetic disorders in the population within a short period of time. In Holstein Friesian breeding several examples can be found such as brachyspina, bovine leukocyte adhesion deficiency or complex vertebral malformation. Direct genotyping was shown to be very effective towards eradication the diseases from the population, if breeding accounts for it [38]. The availability of large-scale genomic SNP data for breeding populations made the interrogation for occult disorders possible. Such would be characterized by the lack of homozygous cattle, which is a strong indication for recessive lethal mutations. Several haplotypes with this appearance have been detected in HF and for some of those the causative genes have been defined in the past. However, the actual causative mutation for one important, since frequent haplotype (HH5), was still unknown.

More recently a disease has been observed with increasing frequency, characterized by severe diarrhea and growth cessation of calves. Based on the available SNP genotyping data, this was linked to a region on BTA11 and the fact that the affected calves suffer from hypocholesterolemia suggested *APOB* as positional and functional candidate for this haplotype, *i*.*e*. cholesterol deficiency. Very recently the herein described insertion was identically found by another independent group, using a different sequencing strategy as most likely causative variant for the disease [39]. Our findings, in particular the large-scale confirmation in comparison with imputed haplotypes, corroborate those results and there is now a large and convincing body of evidence for the 1.3kbp ERV-2-1-LTR_BT insertion in the *APOB* gene as causative mutation for CD.

Both haplotypes were traced back to founders and for CDH the haplotyping alone was not reliable also due to the existence of the same haplotype without harboring the apparent mutation, leading to unaffected healthy calves with the same haplotype. The addition of pedigree data resulted in a better, but still error prone prediction for the disease compared to the haplotyping approach alone.

Using a selective enrichment of the genomic regions for the haplotypes, we were able to uncover the causative mutations. The advantage of such a strategy as compared to whole genome sequencing is the reduced costs, enabling the investigation of a higher number of samples at higher base coverage. Especially for those haplotypes, lacking of homozygous individuals, this is of high benefit, since a base coverage of >20x is needed for de novo SNP detection, given the underlying hypergeometric read distribution and unavoidable sequencing errors.

For HH5 on BTA9, we were able to identify a ~138kb deletion harboring the entire *TFB1M* gene. This deletion most likely occurred by a meiotic homologous recombination/deletion process in the single-strand phase [34] between two bovine long interspersed nuclear elements, L1 and BovB. Both together account for about 22% of the bovine genome [40] and we show a deletion between two homologous regions, with a high thermodynamic ds-stability. The deleted non-redundant protein is necessary for ribosomal function [41], and—in line with the suggested lethality of HH5—*TFB1M*^-/-^ mice are showing embryonic death at day 8.5 [37]. Furthermore cell specific knockout confirmed severe lethal impact on the mammalian cells due to the complete loss of protein translation in mitochondria [37]. Given the fact that no other gene is located in the deleted region, together with aforementioned data and the high segregation with HH5 imputation, these results can serve as a prove of evidence for this deletion to be the cause of HH5. The detection rate of HH5 by imputing is approximately 91%, when compared to the direct mutation assay by PCR.

For CDH on BTA11, an insertion of an endogenous retrovirus type ERV2-1, truncated to the LTR into exon 5 of the *APOB* gene was uncovered, leading to a stop codon shortly after the insertion point. Only one region was found that fulfills the criteria for an active ERV being a sequence in order of: LTR(U2/r/U5)_full ERV(gag/pol/env)_LTR(U3/r/U5) at BTA24:12811498–12820655. Therefore it can be speculated that this is most likely the source of the solo LTR insertion. The presence of so called “solo LTRs” is thought to be based on a homologous recombination between the 5’ and 3’ LTR that are identical and present in phase on both sides of the ERV [32, 42]. Such ERV-LTR integrations can have several consequences, based on their genomic positions. It can–as seen here—disrupt open reading frames, but also the opposite is know, integration into a gene promoter can result in overexpression of RNA [32]. These findings and those to come, beg the question on the role of repetitive elements in shaping evolutionary events through non-lethal alterations.

As pathobiochemical consequence of this insertion, the resulting truncated APOB protein consists of less than 140 amino acids. It can be definitely assumed that the truncated APOB is not functionally active, as the normal size of the human liver variant ApoB100 has a length of 4,500 amino acids and the intestine variant ApoB48 about half this length. The truncation will presumably result in an inability of chylomicron excretion from intestinal cells. This is known from the human equivalent of APOB deficiency, also depending on the length of the truncated protein. Interestingly if the truncation point is close to the natural intestinal variant stop codon, even hyperchylomicronemia was reported [22]. In contrast, in the case of the bovine *APOB* mutation reported herein, the very early premature stop leads to a non-functional intestinal variant as well, with severely impaired fat uptake in the gut. Consequently the synthesis of LDL and VLDL particles in the liver is affected the same way, which all together leads to a severe hypocholesterolemia. However, CDH is not strictly lethal as it can be ameliorated by dietary measures. On the other hand, blood cholesterol homeostasis is affected by a plethora of factors, including cholesterol intake, infectious diseases and other severe disorders resulting in low blood cholesterol. This leads to substantial diagnostic difficulties of CDH using biochemical parameters alone, *e*.*g*. plasma cholesterol levels. Therefore, the only direct diagnosis should be based on the detection of the causative mutation. We have determined close to 12% false negative results in the PCR confirmed group when SNP haplotyping together with pedigree data was used for diagnosis of CDH, which is the current routine approach.

The high false negative rate for both HH5 and CDH seems to be mainly due to recent haploblock breaks, which we assume have likely happened in several instances, when inspecting the SNP genotype data of the false negative cattle. Such haplotype breaks can be predicted to occur in the population over time. The second source of false negative haplotyping is the noise rate of SNP-chip technology, since miscalls of loci occurs unavoidably to a certain extend [43]. The consequences of false negative results can be extreme in modern breeding. One good example is one single BLAD (Bovine Leukocyte Adhesion Deficiency) mistyped sire in the late 1990s, which was used in high frequency for insemination. As a consequence the allelic frequency of the disease increased again from 3% to 5.6% in the German HF population during the next two years [38].

Imputation from large-scale population genomics data has its strength in detecting unknown recessive lethal haplotypes and to bridge the time until the causing mutation is uncovered. Given the biologic event of free recombination [44, 45], it can never perfectly segregate, which we show herein. A higher density of inferred loci around the causal mutation could counteract this for a certain time (number of meioses). That said, the carrier frequency was significantly underestimated by haplotyping for both HH5 and CDH so far. The detailed inspection of discrepancies between imputing results and occurrence of the causative mutation can help to re-adjust the haplotyping for the use on historic SNP-data. The avoidance of false wild-type calls due to technical failures seems less manageable. Nevertheless the inclusion of gene call likelihood data that are provided for each genotype interrogated with an array technology in the imputing pipelines of the database providers would be an option to recommend. Overall for highest accuracy, it is advisable to directly interrogate the causative mutation by independent methods or to include probes for the causing variant to the SNP arrays used for mass SNP genotyping in cattle nowadays. This is in particular important for CDH, since it has—apart from the economical impact of the other lethal haplotypes, which lead to prenatal death–a substantial ethical and animal welfare impact, due to the fact that calves with substantial disease burden and with an 80% lethality within the first year of life [14] are born.

## Supporting Information

S1 Fig HH5 diagnostic PCR. Wild-type: Left lane/red curve; HH5 carrier: right lane/blue curve.
(TIF)

S2 Fig CDH diagnostic PCR. Wild-type: left lane/pink curve, CDH (homozygous.): middle lane/red curve; CDH carrier: right lane/red curve.
(TIF)

S3 Fig Positioning of softclips in enriched CDH Region. Chromosomal positions of softclips were extracted from BAM-files with the condition of being at least 20bp in length, counted in 200bp bins and the delta between CDH and controls was calculated. The two high differences (blue dots) at ~75Mb did a) not converge into one single chromosomal position and were b) not found in each CDH sample. The difference at 77.9Mb (red dot) fulfilled both conditions, positioning the insertion point to BTA11:77,958,995 (UMD3.1).
(TIF)

S4 Fig Deletion of *TFB1M* in HH5 cattle. Read counts were summed in 1kbp bins, normalized to total reads followed by coverage calculation. The data of the HH5 group was converted into copy number values, using the control groups as basis (assuming a copy number of 2 for each bin). Data are given for the HH5 group as log2 ratio, where a value of -1 indicates a one copy loss. Positions are UMD3.1.
(TIF)

S1 Table BAC clones covering the inferred HH5 haplotype region. (DOCX)

S2 Table BAC clones covering the inferred CDH haplotype region. (DOCX)

S3 Table Primers used for HH5 deletion and CDH insertion genotyping. (DOCX)
