# Correction: The Holstein Friesian Lethal Haplotype 5 (HH5) Results from a Complete Deletion of *TBF1M* and Cholesterol Deficiency (CDH) from an ERV-(LTR) Insertion into the Coding Region of *APOB*

**DOI:** 10.1371/journal.pone.0157618

**Published:** 2016-06-09

**Authors:** Ekkehard Schütz, Christin Wehrhahn, Marius Wanjek, Ralf Bortfeld, Wilhelm E. Wemheuer, Julia Beck, Bertram Brenig

The gene name “*TFB1M*” is misspelled in the title. The correct title is: The Holstein Friesian Lethal Haplotype 5 (HH5) Results from a Complete Deletion of *TFB1M* and Cholesterol Deficiency (CDH) from an ERV-(LTR) Insertion into the Coding Region of *APOB*. The correct citation is: Schütz E, Wehrhahn C, Wanjek M, Bortfeld R, Wemheuer WE, Beck J, et al. (2016) The Holstein Friesian Lethal Haplotype 5 (HH5) Results from a Complete Deletion of *TFB1M* and Cholesterol Deficiency (CDH) from an ERV-(LTR) Insertion into the Coding Region of *APOB*. PLoS ONE 11(4): e0154602. doi:10.1371/journal.pone.0154602
