# Supplementary figures and images for: The Holstein Friesian Lethal Haplotype 5 (HH5) Results from a Complete Deletion of TBF1M and Cholesterol Deficiency (CDH) from an ERV-(LTR) Insertion into the Coding Region of APOB
Source: PLoS One. 2016 Apr 29;11(4):e0154602. doi: 10.1371/journal.pone.0154602 (PMC4851415; doi:10.1371/journal.pone.0154602)

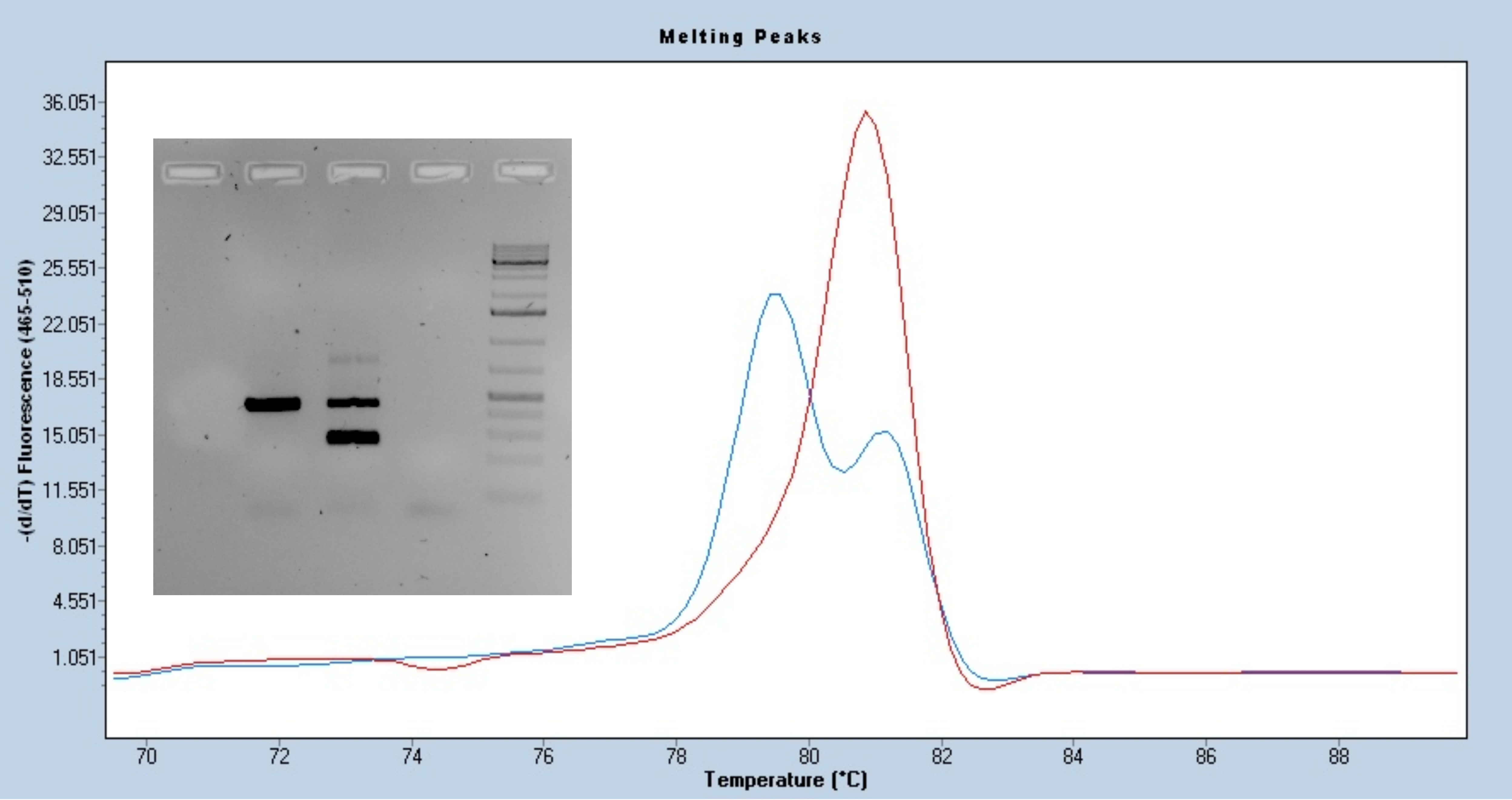

Supplement: S1 Fig — Wild-type: Left lane/red curve; HH5 carrier: right lane/blue curve. (TIF) [file pone.0154602.s001.tif]

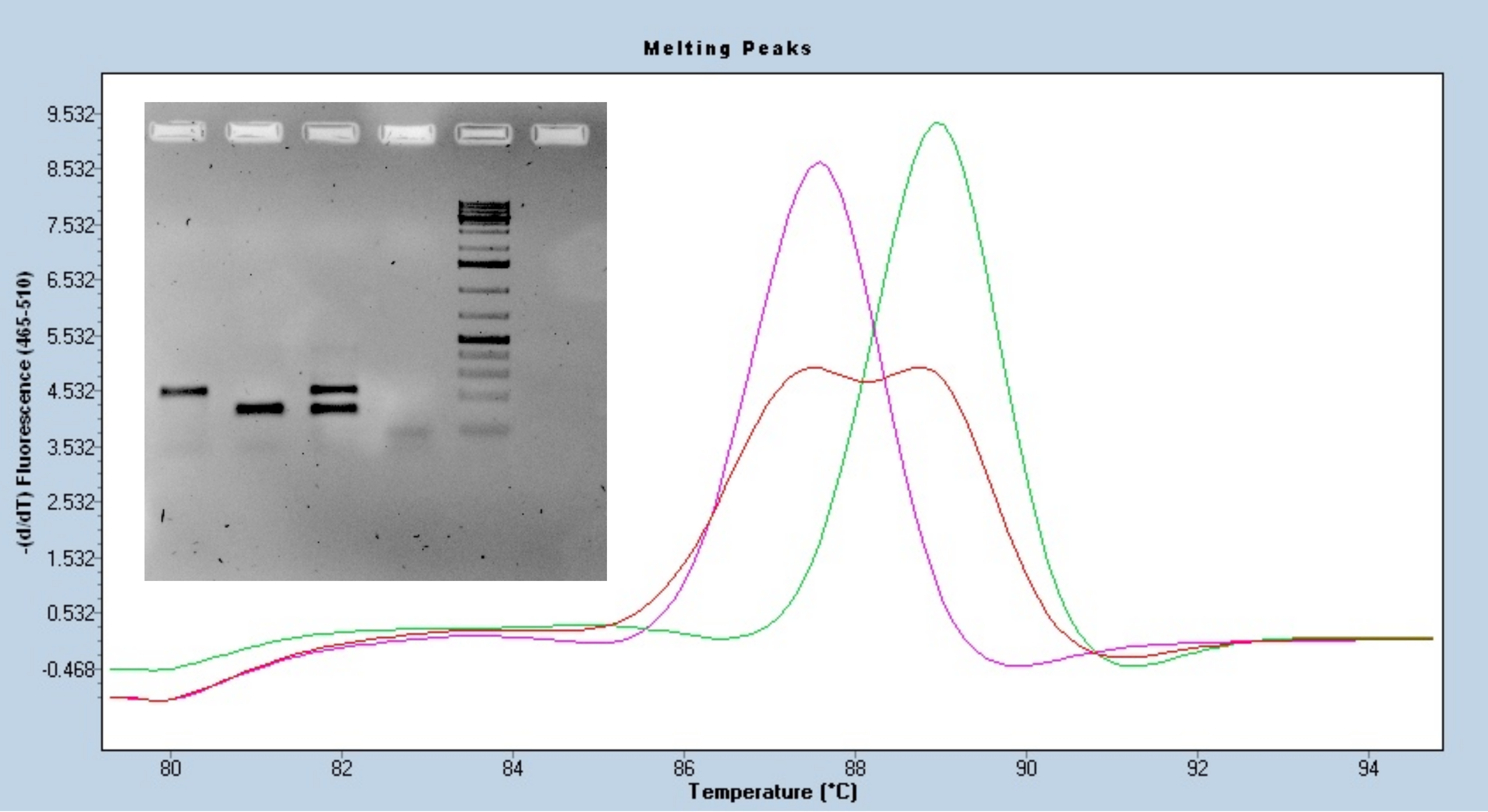

Supplement: S2 Fig — Wild-type: left lane/pink curve, CDH (homozygous.): middle lane/red curve; CDH carrier: right lane/red curve. (TIF) [file pone.0154602.s002.tif]

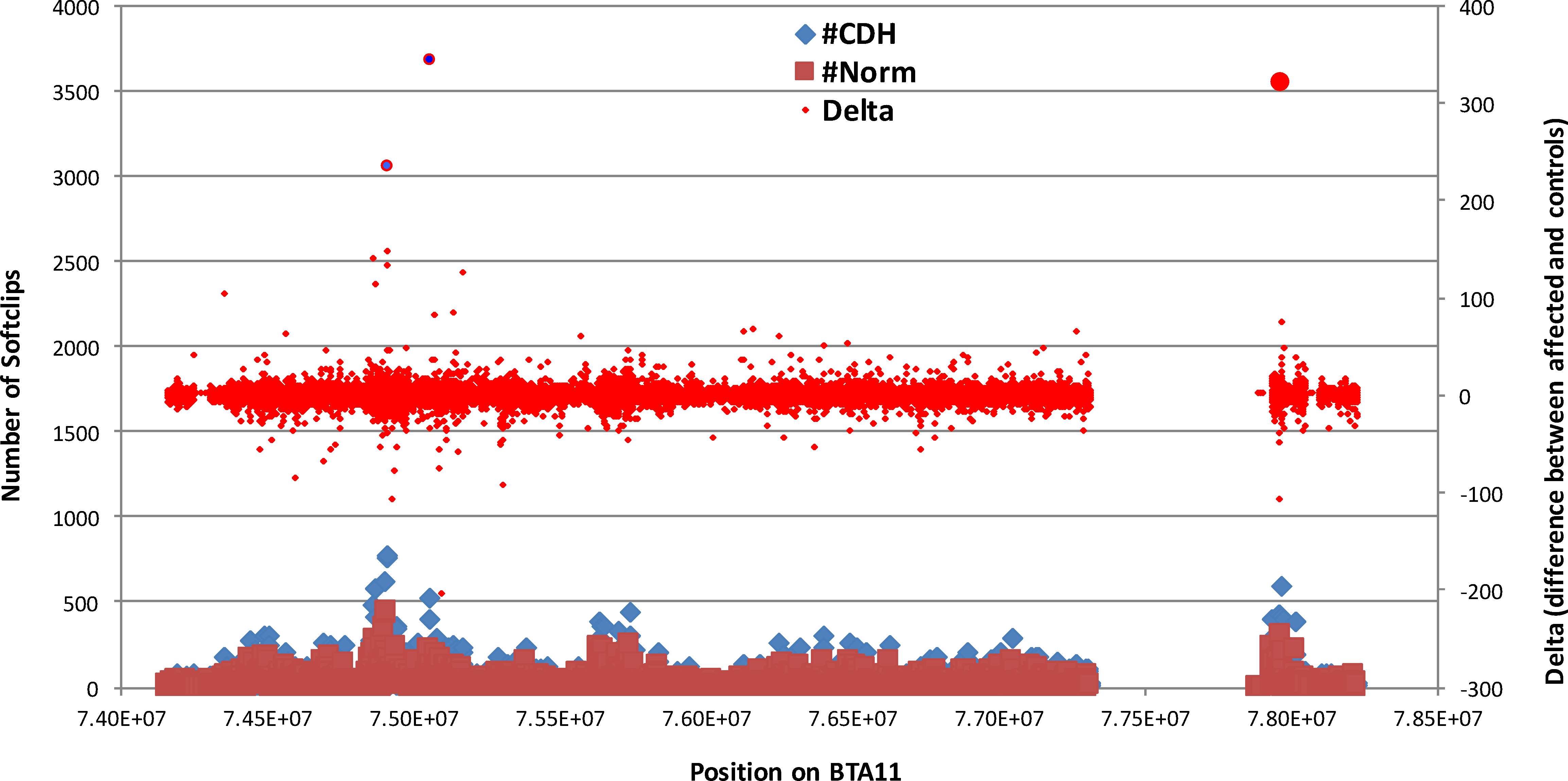

Supplement: S3 Fig — Chromosomal positions of softclips were extracted from BAM-files with the condition of being at least 20bp in length, counted in 200bp bins and the delta between CDH and controls was calculated. The two high differences (blue dots) at ~75Mb did a) not converge into one single chromosomal position and were b) not found in each CDH sample. The difference at 77.9Mb (red dot) fulfilled both conditions, positioning the insertion point to BTA11:77,958,995 (UMD3.1). (TIF) [file pone.0154602.s003.tif]

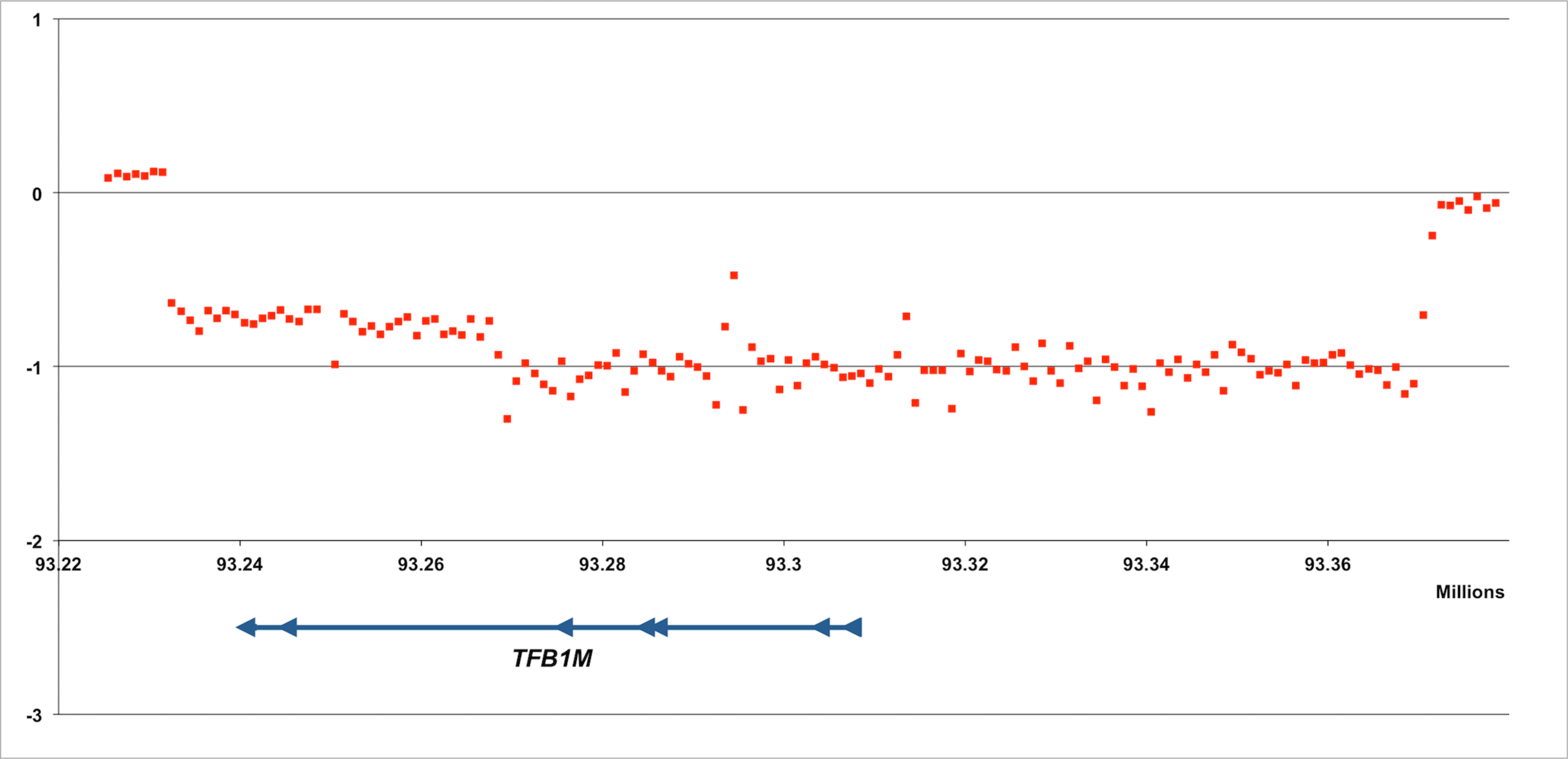

Supplement: S4 Fig — Read counts were summed in 1kbp bins, normalized to total reads followed by coverage calculation. The data of the HH5 group was converted into copy number values, using the control groups as basis (assuming a copy number of 2 for each bin). Data are given for the HH5 group as log2 ratio, where a value of -1 indicates a one copy loss. Positions are UMD3.1. (TIF) [file pone.0154602.s004.tif]
