# Supplementary material for: The Holstein Friesian Lethal Haplotype 5 (HH5) Results from a Complete Deletion of TBF1M and Cholesterol Deficiency (CDH) from an ERV-(LTR) Insertion into the Coding Region of APOB
Source: PLoS One. 2016 Apr 29;11(4):e0154602. doi: 10.1371/journal.pone.0154602 (PMC4851415; doi:10.1371/journal.pone.0154602)
Supplement: S1 Table — (DOCX) [file pone.0154602.s005.docx]

**S1 Table. BAC clones covering the inferred HH5 haplotype region.**

| **CloneName** | **Length (bp)** | **Position on BTA9** |
| --- | --- | --- |
| CH240-348A4 | 182,235 | 91,769,489 - 91,951,723 |
| CH240-333J3 | 173,251 | 91,869,734 - 92,042,984 |
| CH240-395A1 | 146,238 | 92,008,610 - 92,154,847 |
| CH240-37I15 | 128,948 | 92,096,511 - 92,225,458 |
| CH240-423D18 | 134,628 | 92,220,425 - 92,355,052 |
| CH240-399F7 | 160,167 | 92,286,725 - 92,446,891 |
| CH240-323E6 | 169,558 | 92,398,688 - 92,568,245 |
| CH240-149O13 | 166,810 | 92,565,792 - 92,732,601 |
| CH240-224M15 | 154,604 | 92,729,806 - 92,884,409 |
| CH240-444J13 | 202,424 | 92,856,215 - 93,058,638 |
| CH240-475L22 | 154,998 | 93,047,176 - 93,202,173 |
| CH240-200C19 | 75,984 | 93,192,752 - 93,268,735 |
| CH240-43F3 | 183,232 | 93,251,485 - 93,434,716 |
| CH240-382M15 | 240,666 | 93,412,829 - 93,653,493 |
| CH240-235O4 | 162,043 | 93,616,106 - 93,778,148 |
| CH240-20O20 | 213,328 | 93,751,152 - 93,964,479 |
