# Supplementary material for: The Holstein Friesian Lethal Haplotype 5 (HH5) Results from a Complete Deletion of TBF1M and Cholesterol Deficiency (CDH) from an ERV-(LTR) Insertion into the Coding Region of APOB
Source: PLoS One. 2016 Apr 29;11(4):e0154602. doi: 10.1371/journal.pone.0154602 (PMC4851415; doi:10.1371/journal.pone.0154602)
Supplement: S2 Table — (DOCX) [file pone.0154602.s006.docx]

**S2 Table. BAC clones covering the inferred CDH haplotype region.**

| CloneName | Length (bp) | Position on BTA11  (UMD_3.1) |
| --- | --- | --- |
| CH240-22K10 | 194,955 | 74,158,933 - 74,353,887 |
| RP42-114O17 | 175,733 | 74,353,048 - 74,528,780 |
| CH240-347B1 | 134,378 | 74,471,411 - 74,605,788 |
| CH240-427O22 | 184,475 | 74,581,190 - 74,765,664 |
| CH240-394J2 | 145,054 | 74,742,110 - 74,887,163 |
| CH240-175B10 | 139,251 | 74,839,682 - 74,978,932 |
| RP42-143P16 | 133,084 | 74,951,064 - 75,084,147 |
| CH240-251E7 | 131,213 | 75,049,469 - 75,180,681 |
| RP42-137E19 | 178,720 | 75,172,075 - 75,350,794 |
| CH240-355M5 | 125,361 | 75,337,244 - 75,462,604 |
| CH240-200H20 | 202,224 | 75,458,523 - 75,660,746 |
| RP42-127E9 | 127,795 | 75,634,505 - 75,762,299 |
| CH240-303E22 | 154,863 | 75,732,355 - 75,887,217 |
| CH240-460D7 | 157,647 | 75,793,607 - 75,951,253 |
| CH240-436F21 | 182,392 | 75,930,673 - 76,113,064 |
| RP42-137F8 | 135,021 | 76,112,902 - 76,247,922 |
| CH240-342P3 | 167,065 | 76,234,055 - 76,401,119 |
| CH240-95I11 | 147,431 | 76,399,714 - 76,547,144 |
| RP42-14H23 | 176,972 | 76,464,268 - 76,641,239 |
| CH240-379O5 | 168,292 | 76,624,169 - 76,792,460 |
| CH240-199D11 | 213,251 | 76,764,895 - 76,978,145 |
| RP42-45G15 | 171,196 | 76,973,061 - 77,144,256 |
| CH240-125H22 | 203,803 | 77,112,124 - 77,315,926 |
| CH240-472A14 | 194,888 | 77,879,425 - 78,074,311 |
| CH240-6J13 | 112,020 | 77,935,353 - 78,047,371 |
| CH240-445J22 | 253,442 | 77,974,767 - 78,228,207 |
