# Supplementary material for: The Holstein Friesian Lethal Haplotype 5 (HH5) Results from a Complete Deletion of TBF1M and Cholesterol Deficiency (CDH) from an ERV-(LTR) Insertion into the Coding Region of APOB
Source: PLoS One. 2016 Apr 29;11(4):e0154602. doi: 10.1371/journal.pone.0154602 (PMC4851415; doi:10.1371/journal.pone.0154602)
Supplement: S3 Table — (DOCX) [file pone.0154602.s007.docx]

**S3 Table. Primers used for HH5 deletion and CDH insertion genotyping.**

| **Name** | **Sequence (5’-3’)** | **Chromosomal position (UMD_3.1)** | **Amplicon Length WT/MUT**^a)^ **(bp)** | **Melting Peak WT/MUT (°C)** |
| --- | --- | --- | --- | --- |
| HH5-TFB1M.F | AGATATGCTAAAGTTTACCTAGAAGAA | BTA9: 93,371,172 - 93,371,146 |  |  |
| HH5-TFB1M.WT.R | CTGAAGCTCCATTCTGAGTCAT | BTA9: 93,370,731 - 93,370,752 | 442/-- | 80.5/-- |
| HH5-TFB1M.Del.R | TGCTCTATGAATTTTGTGAATGGT | BTA9: 93,232,580 - 93,232,603 | 138,593*/256 | --/79.0 |
| CD-APOB.e3.WT.F | CTGCAAAGCCACCTAGCCTA | BTA11: 77,958,900 - 77,958,919 |  |  |
| CD-APOB.e3.WT.R | GCCTCTTCTGTTTCTGGGGG | BTA11: 77,959,105 - 77,959,086 | 206/1493^b)^ | 86.6/-- |
| CD-APOB.e3.Ins.R | TCACGAGTGGAATGCCTCAC |  | --/104 | --/88.7 |
| LongRange_APOB.F | CTGCAAAGCCACCTAGCCTA | BTA11: 77,958,900 - 77,958,919 |  | n.a. |
| LongRange_APOB.R | TGCCCCTCTTGATGTTGAGG | BTA11: 77,958,045 - 77,959,065 | 166/1461 | n.a. |

a) WT= wild type allele; MUT= mutant allele; b) under the given assay conditions, these amplicons are not synthesized
